# Oxidative stress-responsive apoptosis inducing protein (ORAIP) plays a critical role in cerebral ischemia/reperfusion injury

**DOI:** 10.1038/s41598-019-50073-8

**Published:** 2019-09-18

**Authors:** Masao Kishimoto, Jun Suenaga, Hajime Takase, Kota Araki, Takako Yao, Tsutomu Fujimura, Kimie Murayama, Ko Okumura, Ryu Ueno, Nobuyuki Shimizu, Nobutaka Kawahara, Tetsuya Yamamoto, Yoshinori Seko

**Affiliations:** 1 Department of Neurosurgery, Yokohama City University Graduate School of Medicine, Yokohama, Japan; 2 Division of Cardiovascular Medicine, The Institute for Adult Diseases, Asahi Life Foundation, Tokyo, Japan; 3 Laboratory of Bioanalytical Chemistry, Tohoku Medical and Pharmaceutical University, Sendai, Japan; 4 Division of Proteomics and Biomolecular Science, BioMedical Research Center, Graduate School of Medicine, Juntendo University, Tokyo, Japan; 5 Department of Biofunctional Microbiota, Juntendo University School of Medicine, Tokyo, Japan

**Keywords:** Neuro-vascular interactions, Stroke

## Abstract

Oxidative stress is known to play a critical role in the pathogenesis of various disorders, especially in ischemia/reperfusion (I/R) injury. We identified an apoptosis-inducing humoral factor and named this novel post translationally modified secreted form of eukaryotic translation initiation factor 5A (eIF5A) “oxidative stress-responsive apoptosis inducing protein” (ORAIP). The purpose of this study was to investigate the role of ORAIP in the mechanisms of cerebral I/R injury. Hypoxia/reoxygenation induced expression of ORAIP in cultured rat cerebral neurons, resulting in extensive apoptosis of these cells, which was largely suppressed by neutralizing anti-ORAIP monoclonal antibody (mAb) *in vitro*. Recombinant-ORAIP induced extensive apoptosis of cerebral neurons. Cerebral I/R induced expression of ORAIP in many neurons in a rat tandem occlusion model *in vivo*. In addition, we analyzed the effects of intracerebroventricular administration of neutralizing anti-ORAIP mAb on the development of cerebral infarction. Cerebral I/R significantly increased ORAIP levels in cerebrospinal fluid. Treatment with intracerebroventricular administration of neutralizing anti-ORAIP mAb reduced infarct volume by 72%, and by 55% even when started after reperfusion. These data strongly suggest that ORAIP plays a pivotal role and will offer a critical therapeutic target for cerebral I/R injury induced by thrombolysis and thrombectomy for acute ischemic stroke.

## Introduction

Stroke is the worldwide leading cause of severe morbidity and mortality in the elderly and causes 9% of all deaths around the world, representing the second most common cause of death after ischemic heart disease. Approximately 80% of all strokes are ischemic^1,2^. For acute ischemic stroke (AIS), stand-alone or combined therapies using intravenous thrombolysis with tissue plasminogen activator (tPA)^3,4^ and mechanical thrombectomy using an aspiration device and stent retriever have demonstrated rapid, safe, and effective recanalization^5–15^. However, reperfusion increases the production of reactive oxygen species (ROS) which in turn leads to oxidative stress associated injury, lipid peroxidation, protein oxidation, and DNA damage in cells, resulting in disruption of the blood-brain barrier (BBB) and edema in the brain tissues^16,17^.

Oxidative stress is known to play a critical role in the pathogenesis of various disorders, including atherosclerosis^18^, aging^19^, and, in particular, ischemia/reperfusion (I/R) injury. Until recently, most of the literature has proposed ROS as the key mediator of oxidative stress-induced cell injury^20–22^. However, large-scale clinical trials of antioxidants (including vitamins and free radical scavengers) have proven disappointing in terms of improving outcomes for cardiovascular and cerebrovascular diseases in humans^23–27^. Although there are many reasons why antioxidants have failed in clinical trials, such unexpected findings have also raised the possibility that unknown mechanisms other than ROS may be mediating oxidative stress-induced cell injury.

We previously reported that a novel post-translationally modified eukaryotic translation initiation factor 5 A (eIF5A) is rapidly secreted from cardiac myocytes in response to hypoxia/reoxygenation and then acts as the apoptosis-inducing factor in an autocrine fashion^28^. We refer to this novel tyrosine-sulfated secreted form of eIF5A as “oxidative stress-responsive apoptosis inducing protein” (ORAIP)^28^. We found that the secretion of ORAIP from cells specifically occurs in response to oxidative stresses, such as I/R, ultraviolet burn^28^, ionizing radiation, heat shock, and blood acidification^29^, and then induces apoptosis in target cells such as cardiac myocytes. We also reported that plasma ORAIP levels were significantly elevated in chronic diseases such as chronic kidney disease, diabetes mellitus, and atrial fibrillation, in which oxidative stress is critically involved in the pathogenesis^30–32^. As we previously reported, ORAIP activates initiator caspases through the MAPK pathway and Jak/STAT pathway^28^. The precise mechanism by which the putative ORAIP receptor (ORAIP-R) transduces apoptotic signaling through these pathways remains unclear and under investigation.

The purpose of this study was to investigate whether the same mechanism involved in myocardial I/R injury is involved in cerebral I/R injury. Here, we show that ORAIP plays a pivotal role in a rat model of cerebral I/R injury, offering a novel efficacious prevention therapy against cerebral I/R injury.

## Results

### Expression of ORAIP in cerebral neurons subjected to hypoxia/reoxygenation *in vitro*

**Figure 1:**
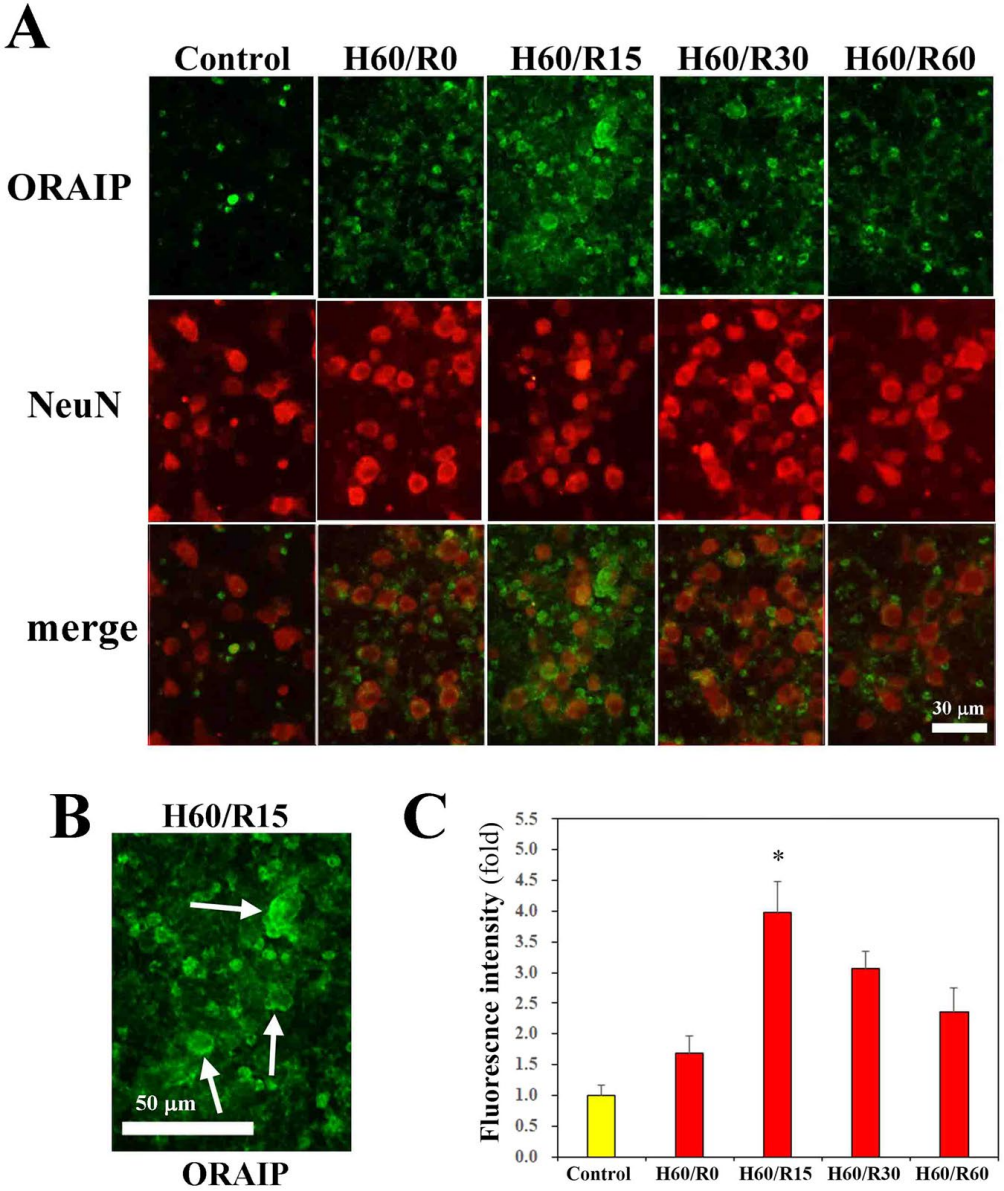
Expression of ORAIP in cerebral neurons subjected to hypoxia/reoxygenation *in vitro*. (**A**) Immunofluorescent localization of ORAIP proteins in cultured cerebral neurons subjected to hypoxia/reoxygenation as determined by double-immunostaining with anti-ORAIP mAb (YSP5-45-36, upper panels) and anti-NeuN antibody (ABN78, middle panels). Lower panels show the merged images. Bar, 50 μm. (**B)** Higher magnification of H60/R15 cells shows expressed ORAIP proteins as many granular stainings in the peripheral cytoplasm of neurons (arrows). (**C**) Quantitative analysis shows that expression of ORAIP peaked at 15 min after reoxygenation. ([mean ± s.d.] n = 4, *P < 0.01 vs, H60/R30, *P < 0.001 vs Control, Hypox 20/Reoxy 0 and 60). (Dunnett’s multiple comparison test).

To investigate whether hypoxia/reoxygenation activated cerebral neurons and induced expression of ORAIP, we first examined Western blots for phospho-extracellular signal-regulated kinase (ERKs), one of the most sensitive markers of ORAIP-induced signaling^28^, in cerebral neurons subjected to hypoxia/reoxygenation *in vitro*. We found that hypoxia/reoxygenation significantly activated ERKs, reaching a peak level by 30 min after reoxygenation (Supplemental Fig. 1 in the online-only Data Supplement). We then examined the induction of ORAIP in cerebral neurons subjected to hypoxia/reoxygenation by double-immunostaining for ORAIP and the neuron-specific neuronal nuclei (NeuN) antigen, showing that most cells were neurons (Fig. 1A). Cerebral neurons under normoxic conditions only weakly expressed ORAIP, whereas neurons subjected to hypoxia/reoxygenation showed clear expression of ORAIP in the form of many granular stainings within the peripheral cytoplasm (Fig. 1B; higher magnification of H60/R15, arrows). ORAIP expression peaked at 15 min after reoxygenation (Fig. 1A,C). Immunofluorescence staining with mouse IgG instead of anti-ORAIP mAb as negative controls showed no significant signal (data not shown). These findings were consistent with those previously reported for cardiac myocytes subjected to hypoxia/reoxygenation^28^.

### ORAIP dominantly mediates hypoxia/reoxygenation-induced apoptosis in cerebral neurons *in vitro*

**Figure 2:**
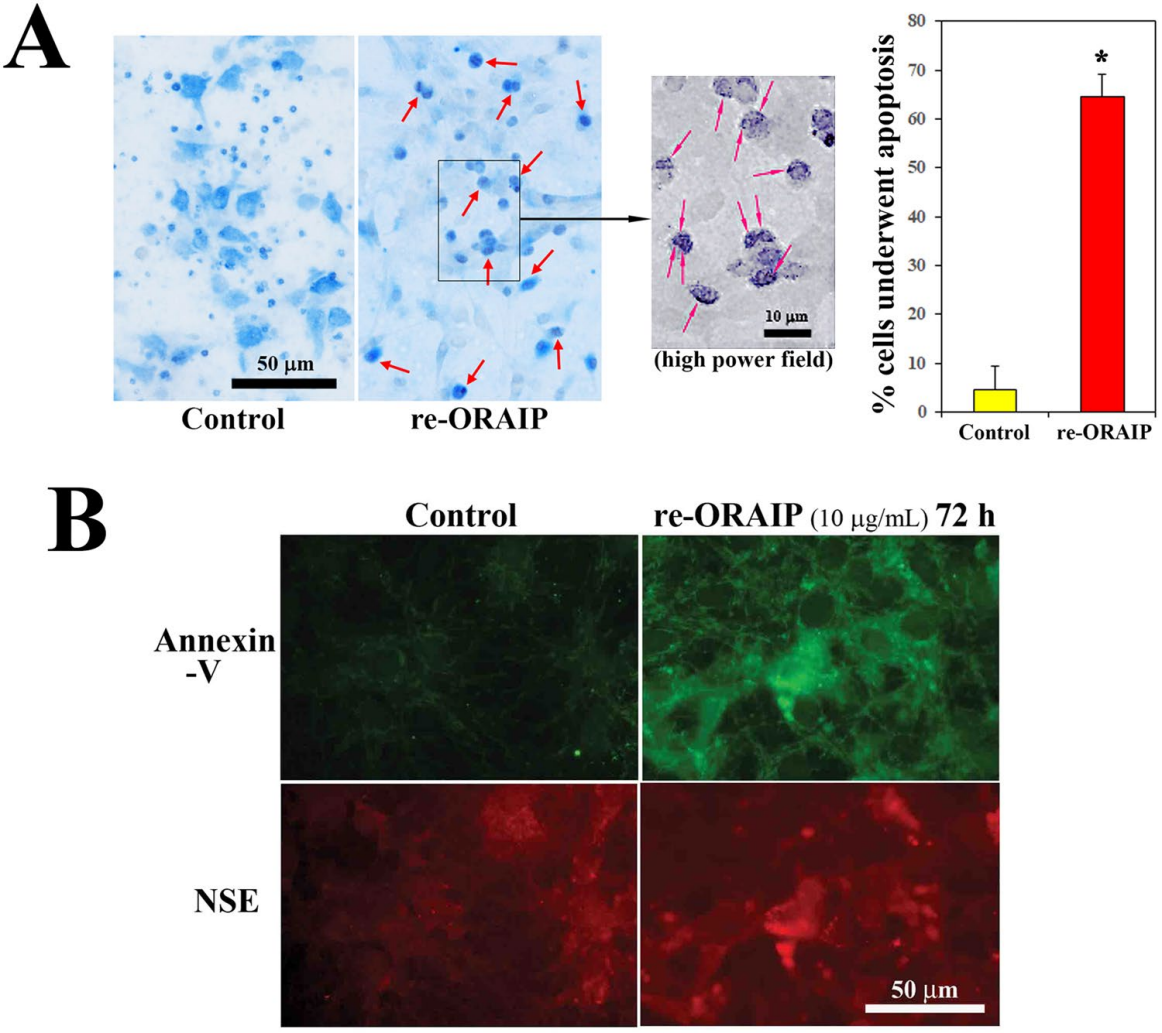
Induction of apoptosis in cerebral neurons by re-ORAIP *in vitro*. (**A)** Induction of apoptosis in cerebral neurons as determined by TUNEL staining (brown; red arrows) and NeuN immunostaining (blue). Representative images of control (left panel) and re-ORAIP-treated for 48 h (middle panel). Bar, 50 μm. Higher magnification of re-ORAIP-treated group shows many granular stainings in the nuclei (right panel, red arrows). Graph shows the percentage of apoptotic cerebral neurons, as determined by TUNEL staining, induced by re-ORAIP (10 μg/mL for 48 h). Data are expressed as ([mean ± s.d.], n = 4 each) *P < 0.001 vs. Control (Welch’s *t*-test). (**B)** Induction of apoptosis in cerebral neurons by re-ORAIP (10 μg/mL for 72 h) as determined by double-immunostaining for annexin-V (upper panels, labeled with FITC) and NSE (lower panels, labeled with TRITC). Bar, 50 μm.

**Figure 3:**
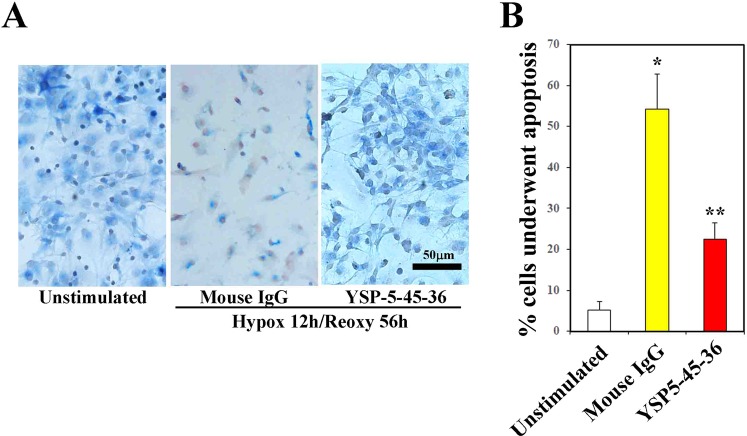
Effects of neutralization with anti-ORAIP mAb on hypoxia (12 h)/reoxygenation (56 h)-induced apoptosis induction in cultured cerebral neurons. (**A**) TUNEL staining (brown) and NeuN immunostaining (blue) of unstimulated cells and cells subjected to hypoxia/reoxygenation with control mouse IgG (0.05 mg/mL) or anti-ORAIP mAb (YSP5-45-36; 0.05 mg/mL). Representative images at 56 h after reoxygenation. (**B**) Percentage of apoptotic cerebral neurons as determined by TUNEL staining at 56 h after reoxygenation. *P < 0.0001 vs. mouse IgG ([mean ± s.e.m.], n = 4 each) (Tukey-Kramer method).

Next, to investigate whether secreted ORAIP from cerebral neurons in response to hypoxia/reoxygenation induced apoptosis in neurons, we examined whether re-ORAIP induced neural apoptosis by double-immunostaining for terminal deoxynucleotidyl transferase-mediated deoxyuridine triphosphate nick-end labeling (TUNEL) and NeuN (Fig. 2A), as well as double-immunostaining for neuron-specific enolase (NSE) and annexin-V (Fig. 2B). TUNEL staining showed that re-ORAIP markedly induced apoptosis in neurons within 48 h (Fig. 2A; middle panels, red arrows). Induction of apoptosis in neurons by treatment with re-ORAIP for 72 h was further confirmed by annexin-V staining (Fig. 2B). Furthermore, to confirm whether autocrine secretion of ORAIP induced apoptosis in cerebral neurons in response to hypoxia/reoxygenation, we examined the effects of neutralizing anti-ORAIP monoclonal antibody (mAb) (YSP5-45-36) on the induction of apoptosis. Double-immunostaining for TUNEL and NeuN showed that hypoxia (12 h) and reoxygenation (56 h) induced apoptosis in 54% of neurons (with mouse IgG), and this apoptosis was 60% suppressed by neutralizing anti-ORAIP mAb, indicating that apoptosis induction in neurons was predominantly mediated by ORAIP in an autocrine fashion (Fig. 3A,B).

### Expression of ORAIP in cerebral tissues subjected to I/R *in vivo*

**Figure 4:**
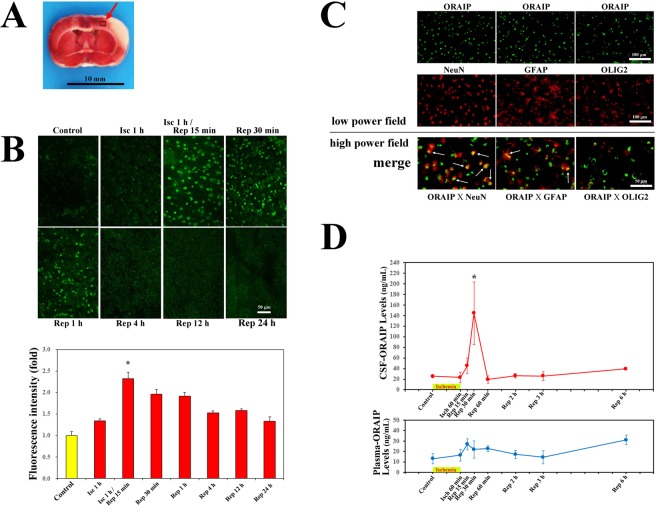
Expression of ORAIP in cerebral tissues and CSF- and plasma-ORAIP concentrations in rats subjected to cerebral I/R *in vivo*. (**A**) Red arrow indicates the penumbra region in a cross-section of cerebral tissue. (**B**) Immunofluorescent localization of ORAIP proteins in cerebral penumbra tissue samples (panel A, red arrow) from control rats (before ischemia) and rats subjected to ischemia/reperfusion *in vivo*. Bar, 50 μm. Quantitative analysis shows that expression of ORAIP peaked at 15 min after reperfusion. ([mean ± s.e.m.] n = 4, *P = 0.012 vs, Isc 1 h/Rep 1 h, *P = 0.026 vs Isc 1 h/Rep 30 min, *P < 0.0001 vs, Control, Isc 1 h, Isc 1 h/Rep 4 h, 12 h, and 24 h. (Dunnett’s multiple comparison test). (**C**) Double-immunostaining of cerebral penumbra tissue samples (panel A; red arrow) from rats subjected to ischemia (60 min)/reperfusion (15 min) for ORAIP and cerebral neural cell markers such as NeuN, GFAP, and OLIG2. Bar, 50 μm. (**D**) Time course of CSF- and plasma-ORAIP levels ([mean ± s.e.m.] ng/mL) in rats subjected to cerebral I/R *in vivo*. CSF-ORAIP levels (upper panel) were significantly increased at 30 min after reperfusion (Rep) (n = 6) compared with normal and 60 min of ischemia (Isch). *P = 0.0289 vs. normal (n = 5). *P = 0.0205 vs. 60 min of ischemia (n = 6) (Dunnett’s multiple comparison test). No significant differences in plasma-ORAIP levels were evident among each time point (lower panel).

Figure 4B shows the expression of ORAIP in tissue samples from the cerebral penumbra (Fig. 4A, red arrow) from control rats (before ischemia) and rats subjected to cerebral ischemia (60 min) with reperfusion *in vivo*. In control rats and rats subjected to ischemia (60 min) alone, barely any ORAIP expression was detected in penumbral tissues. In contrast, ischemia (60 min) with reperfusion (15 min) clearly increased ORAIP expression in many cerebral cells (Fig. 4B). The expression of ORAIP decreased 30 min after reperfusion and returned to almost the control level by 4 h after reperfusion. Double-immunostaining of tissue samples from the cerebral penumbra for ORAIP and cerebral neural cell markers such as NeuN, glial fibrillary acidic protein (GFAP), and oligodendrocyte transcription factor 2 (OLIG2) (Fig. 4C) showed that many neurons (approximately 60%) and a proportion of astrocytes (approximately 10%) expressed ORAIP, whereas almost no oligodendrocytes expressed ORAIP (Fig. 4C, lower panels). To confirm that ORAIP molecules were truly secreted from cerebral tissues subjected to I/R, we measured the ORAIP concentration in the cerebrospinal fluid (CSF) and plasma by enzyme-linked immunosorbent assay (ELISA). No significant changes in CSF levels of ORAIP were observed between the control condition (before ischemia) and after 60 min of ischemia (immediately before reperfusion) (Fig. 4D, upper panel). However, the concentration of ORAIP in the CSF increased and peaked at 144.6 ± 58.9 ng/mL (mean ± s.e.m., n = 6) at 30 min after reperfusion (Rep 30 min), representing a significant increase compared with that in both the normal condition (25.5 ± 2.8 ng/mL, n = 5, *P* = 0.0289) and after 60 min of ischemia alone (Isch) (immediately before reperfusion: 23.3 ± 9.8 ng/mL, n = 6, *P* = 0.0205), and then decreased to the control level from 60 min after reperfusion. No significant difference in ORAIP levels in the CSF was evident between 15 min and 30 min after reperfusion (Fig. 4D, upper panel). In contrast, plasma levels of ORAIP increased slightly and peaked at 15 min after reperfusion (Rep 15 min) (26.8 ± 5.4 ng/mL, n = 5). However, no significant differences were evident among the time points (Fig. 4D, lower panel).

### *In vivo* administration of anti-ORAIP mAb suppresses cerebral I/R injury

**Figure 5:**
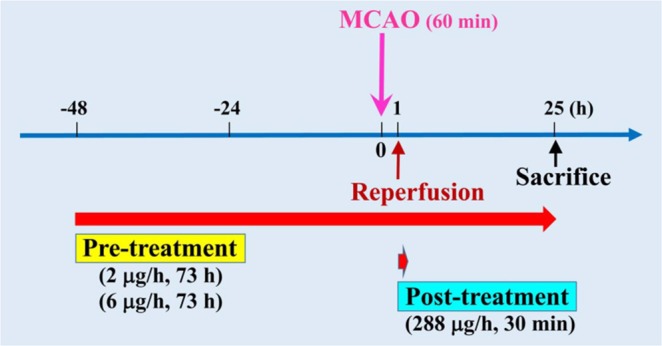
*In vivo* study design and schedule for cerebral I/R and administration of anti-ORAIP mAb.

**Figure 6:**
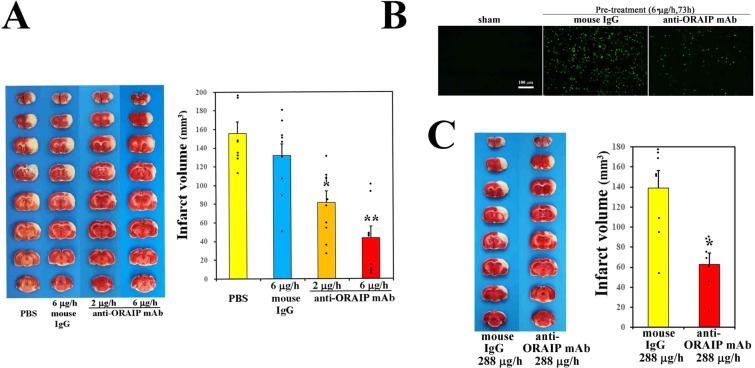
Neutralization of ORAIP suppresses cerebral I/R injury *in vivo*. (**A**) Pre-treatment with anti-ORAIP neutralizing mAb (YSP5-45-36) significantly reduced cerebral I/R injury. Representative cross-sections of cerebrum from the PBS-treated group (n = 8), mouse IgG-treated group (6 μg/h for 73 h, drip infusion), and anti-ORAIP mAb (YSP5-45-36)-treated group (either 2 μg/h or 6 μg/h for 73 h, drip infusion). Infarct volumes in the anti-ORAIP mAb-treated groups (2 μg/h, n = 10; 6 μg/h, n = 9) were significantly smaller than those in the mouse IgG-treated group (2 μg/h, n = 10, *P = 0.0365; 6 μg/h, n = 9, **P = 0.000174) (Tukey-Kramer method). (**B)** TUNEL staining of penumbra tissue samples (see Fig. 4A) from sham-operated, control mouse IgG-treated, and anti-ORAIP mAb-treated (pre-treatment) rats subjected to ischemia (60 min)/reperfusion (24 h). (**C**) Post-treatment with anti-ORAIP neutralizing mAb (YSP5-45-36) significantly reduced cerebral I/R injury. Representative cross-sections of cerebrum from the group treated with mouse IgG (288 μg/h for 30 min, drip infusion; n = 8) and anti-ORAIP mAb (YSP5-45-36)-treated group (288 μg/h for 30 min, drip infusion; n = 7). Infarct volume was significantly smaller in the anti-ORAIP mAb-treated group than in the mouse IgG-treated group (*P = 0.0033) (Welch’s *t*-test).

Because neutralizing anti-ORAIP mAb (YSP5-45-36) dominantly suppressed hypoxia/reoxygenation-induced apoptosis in cerebral neurons *in vitro* (Fig. 3), we investigated the effects of *in vivo* administration of anti-ORAIP mAb on cerebral I/R injury. First, to deliver anti-ORAIP mAb into cerebral tissues completely, we started drip infusion of the mAb through an intraventricular catheter 48 h before the onset of ischemia (for 1 h) until 24 h after reperfusion (Pre-treatment, Fig. 5). Treatment with anti-ORAIP mAb (either 2 μg/h or 6 μg/h for 73 h, drip infusion) significantly reduced the infarct volume (81.58 ± 11.25 mm3 [mean ± s.e.m.], n = 10, *P* = 0.0365; and 43.79 ± 12.37 mm3, n = 9, *P* < 0.001, respectively) compared with the control mouse IgG (6 μg/h for 73 h) treatment (132.15 ± 13.85 mm3, n = 9) (Fig. 6A). TUNEL staining of penumbral tissue samples from sham-operated, control mouse IgG-treated, and anti-ORAIP mAb-treated rats showed marked reductions in apoptotic cells (both total cells and neurons) in the anti-ORAIP mAb-treated group compared with that in the control mouse IgG-treated group (Fig. 6B, Supplemental Fig. 3 in the online-only Data Supplement). Administration of anti-ORAIP mAb (288 μg/h for 30 min, drip infusion) started just after reperfusion significantly suppressed the cerebral infarct volume (62.75 ± 11.30 mm3 [mean ± s.e.m.], n = 7, *P* = 0.0033) compared with control mouse IgG (288 μg/h for 30 min) treatment (139.07 ± 17.12 mm3, n = 8) (Fig. 6C), indicating the effectiveness of anti-ORAIP mAb therapy even when administered after the onset of ischemia.

## Discussion

Cerebral neurons, such as cardiac myocytes, demand a large amount of energy and oxygen for their activities^33,34^, making them very sensitive to oxygen concentrations and hence susceptible to I/R injury. The present study demonstrated that *in vivo* administration of a neutralizing anti-ORAIP mAb critically reduced (by approximately 72%) the degree of cerebral infarction, indicating that ORAIP rather than ROS plays a pivotal role in cerebral I/R injury. This effect was similar to of anti-ORAIP mAb observed in myocardial I/R injury^28^. These results suggest that ORAIP might be a common humoral factor among various cell types as the dominant inducer of apoptosis in response to oxidative stresses. The failure of antioxidant therapy to ameliorate cerebral I/R injury supports this possibility^23,25,26^. Although the free-radical ROS scavenger edaravone has been reported to improve neurological recovery after recanalization by tPA therapy in AIS^35,36^, the evidence is weak and has proven inadequate to confirm the effectiveness of that approach against cerebral I/R injury^37,38^.

Hosoo *et al*.^39^ recently reported that intra-arterial injection of nitroxide radical-containing nanoparticles (RNPs) as a novel ROS scavenger after cerebral I/R injury reduced the infarction volume by approximately 30%, which was thought to represent the degree of involvement of ROS. The upstream oxidative stress-sensing mechanism that triggers ORAIP secretion remains unclear but does not appear to be ROS because the time course of ORAIP secretion is much faster than that of ROS generation^40^. The effects of combination therapy using anti-ORAIP and anti-ROS agents may thus be synergistic. In studies using animal models, increased numbers of bioactive peptides and targeting of various mechanisms have been reported to show effectiveness in preventing cerebral I/R injury^41^. However, few such approaches have shown any real success in clinical trials, potentially because the agents must be effective when administered after the onset of ischemia to be of any clinical use. Therefore, further investigation and development of novel bioactive peptides is needed. Prophylactic administration of agents such as A-type cytosine-guanine (CpG) oligodeoxynucleotide (ODN) may be somewhat effective but is not clinically practical^42^. Several other agents, including microRNAs, have been reported to be effective when administered after the onset of ischemia^43–45^. However, these agents were less effective than the anti-ORAIP mAb therapy in the present study, and the mechanisms involved are elusive and require further validation. To estimate the overall contribution of ORAIP and ROS to cerebral I/R injury, evaluation of anti-ORAIP therapy in future clinical trials is needed.

In the present study, to ensure complete delivery of anti-ORAIP mAb into the cerebral tissues, we first performed drip infusion of the mAb for 73 h through an intraventricular catheter started before the onset of ischemia, as the mAb cannot penetrate the BBB under physiological conditions. BBB disruption and permeability after I/R varies depending on the size of the penumbra or infarct core region^16^. We also demonstrated that drip infusion of anti-ORAIP mAb through an intraventricular catheter for 30 min just after reperfusion suppressed cerebral infarction by more than 55%, indicating that the anti-ORAIP therapy can be effective even when administered after the onset of ischemia and, hence, is likely to prove useful in the clinical treatment of AIS. The efficacy of this treatment after ischemia onset seems reasonable because I/R (but not ischemia alone) is known to induce ORAIP secretion^28^. However, intrathecal administration is not clinically useful because of the invasive nature of the technique. We identified a cell-surface receptor for ORAIP (that is, ORAIP receptor [ORAIP-R]; unpublished data) and intend to analyze the extracellular crystal structure of this protein using a synchrotron. Using 3-dimensional structural information, including the (ORAIP/ORAIP-R) binding site, we plan to identify small-molecule compounds that can inhibit (ORAIP/ORAIP-R) binding to protect against ORAIP-mediated cell injury. As such small molecules would be able to pass through the BBB, anti-ORAIP therapy with small-molecule compounds should be effective by intravenous administration even in AIS of small arteries, in which BBB disruption does not occur.

Cerebral I/R induces ORAIP expression on neurons and astrocytes, which constitute neurovascular units in association with endothelial cells^46^. Because interactions among these cells regulate BBB function, neuroprotection by anti-ORAIP therapy may reduce disruption of the BBB. Mechanical thrombectomy requires a therapeutic time window because delayed reperfusion results in critical brain edema or cerebral hemorrhage. Recent clinical studies have shifted to delayed reperfusion when the ischemic core is not very large and the penumbral region dominates^6,7^. Anti-ORAIP mAb therapy might be useful not only in reducing neuronal cell apoptosis, but also in repairing BBB integrity by reducing the apoptosis of cells in the neurovascular unit.

The present study also showed that ORAIP levels in CSF increased significantly and peaked by 30 min after reperfusion compared with those just before reperfusion. The reason that CSF-ORAIP levels appeared to peak later than plasma ORAIP levels in myocardial ischemia/reperfusion (10–15 min after reperfusion)^28^ seems to be that CSF flow was much slower than blood flow. Due to this slow CSF flow and the presence of the BBB, plasma ORAIP levels may not offer a useful marker of cerebral I/R, as suggested in the present study (Fig. 4D). The present results demonstrated that ORAIP plays a dominant role in cerebral I/R injury, as in the myocardial I/R injury reported previously^28^. This observation suggests that ORAIP might be a common apoptosis-inducing ligand among various cell types, as well as under various types of oxidative stress^29^, and appears likely to represent a critical therapeutic target for oxidative stress-induced cell injury. Because stroke is the leading cause of serious long-term disability, anti-ORAIP therapy for AIS may significantly ameliorate patient conditions and provide a great advance in preventing the associated socioeconomic loss. Because 85% of stroke patients do not receive thrombolytic agents clinically, there will be less indication for anti-ORAIP mAb therapy for such patients. However, without thrombolytic therapy, spontaneous reperfusion in the occluded cerebral artery may occur in some patients due to activation of the native fibrinolytic system. Anti-ORAIP mAb therapy will also be useful for such patients.

Our study has some limitations that need to be considered when interpreting the findings. First, we administered anti-ORAIP mAb only via an intraventricular route and did not study intraarterial or intravenous routes because of the potential difficulties in penetration. Further investigation should focus on the potential use of anti-ORAIP mAb via intraarterial or intravenous routes using mAbs engineered to cross the BBB. Second, the study design only examined up to 24 h after reperfusion and did not provide long-term follow-up because ORAIP-mediated apoptosis occurred only transiently after I/R. Supplemental Fig. 9 in the online-only Data Supplement shows preliminary behavioral data, in which we analyzed neurological behavior at 24 h after reperfusion in the pretreatment group. Although there were no significant differences among the groups, this lack of an effect may be because functional recovery is usually observed around day 7 after stroke, and the functional outcome on day 1 was too early to show the effects of the reduction in infarct volume. However, the clinical course after stroke is dynamic, involving phenomena such as the microglial polarization shift^47^, and future behavioral analyses examining several weeks after I/R will be needed to address this issue. We also need to optimize the dose and duration of anti-ORAIP mAb administration after stroke in future clinical trials.

In conclusion, ORAIP seems to predominantly mediate the newly discovered oxidative stress-induced apoptotic pathway. Anti-ORAIP therapy thus merits attention for clinical application over the currently available anti-apoptotic therapies for reperfusion injury induced by thrombolysis and thrombectomy following AIS. Of course, more work is certainly needed, such as investigations of functional and neurological outcomes, long-term outcomes, and multiple models, to prove the potential utility of anti-ORAIP therapy.

## Materials and Methods

### Animal preparation

Male spontaneously hypertensive rats (SHR; Charles River Laboratories, Yokohama, Japan) aged 11–14 weeks (body weight, 266–315 g) at the time of surgery were used in this study. Animals were housed 2–3 per cage under controlled conditions with a 14-h light/10-h dark cycle with lights on at 07:00, temperature controlled at 24 ± 1 °C, and humidity at 55 ± 5% for at least 1 week before surgery. Food and water were available ad libitum before and after surgery. This study was carried out in accordance with the National Institutes of Health Guide for the Care and Use of Laboratory Animals. All animal experiments in this study were approved by the institutional Animal Care and Use Committee of the Yokohama City University (F-A-14-110, F-A-17-047). All surgery was performed under isoflurane anesthesia, and all efforts were made to minimize the number of animals used and their suffering throughout the experimental procedures. Age and body weight, data from blood gas analyses, physiological parameters, and rectal temperatures after reperfusion of animals in pre-treatment experiments are shown in Supplemental Tables 1–4, respectively. Data from post-treatment experiments are shown in Supplemental Tables 5–8, respectively (in the online-only Data Supplement).

### Experimental design

Rats were randomly assigned to sham and transient middle cerebral artery occlusion (tMCAO) groups through the use of a lottery-drawing box. MCAO groups were further randomly divided into 6 groups, including pre-treatment intracerebroventricular (ICV) injection of phosphate-buffered saline (PBS) or normal mouse IgG as control, anti-ORAIP mAb (2 µg/h or 6 µg/h, for 73 h), or post-treatment of IgG or anti-ORAIP mAb (288 µg/h, for 30 min) (Fig. 5).

### ICV injection

Eight hours prior to ICV injection in pre-treatment experiments, an ALZET osmotic pump (model 1003D; Durect, Cupertino, CA, USA) with a 1.0-µL/h flow rate and 100-µL reservoir, and an ALZET brain infusion kit 2 (Durect) were assembled, filled with each injection material, and primed in sterile PBS under aseptic conditions. Forty-eight hours before tMCAO in pre-treatment experiments, rats were anesthetized with isoflurane (5% for induction, 2% for surgery) in a mixture of 90% room air and 10% oxygen, and placed in a stereotaxic apparatus (model DKI 900; DAVID KOPF, Tujunga, CA, USA). A rectal probe of thermometer was inserted and core temperature was maintained at 37.0 ± 0.5 °C during all surgical procedures using a feedback-controlled heating pad (BWT-100; Bio Research Center, Nagoya, Japan). A midline scalp incision was made to expose the injection point, 1.5 mm lateral and 0.8 mm posterior to the bregma in the left hemisphere. A 1.5-mm burr hole was made and the already-prepared Alzet brain infusion kit was placed stereotactically and vertically through a point 3.5 mm beneath the brain surface to the left lateral ventricle. The kit was affixed to skull with glue and the reservoir was placed under the back skin. Rectal temperature was monitored until recovery of the rat from anesthesia.

### Focal cerebral ischemia/reperfusion

Focal cerebral ischemia/reperfusion was elicited by tMCAO. A rat MCAO model was described by Robinson^48^ in 1975, with a modification described by Tamura in 1981^49^. A model creating permanent tandem occlusion of the distal MCA and ipsilateral common carotid artery (MCA/CCAO) was induced by Brint^50^ in 1988. A transient tandem MCA/CCAO model in SHR has been described previously^51,52^, and we followed this method using minor modifications. Briefly, rats were anesthetized with isoflurane (5% for induction, 2% for surgery, 1.5% for maintenance) in a mixture of 90% room air and 10% oxygen. A rectal probe was inserted and core temperature was maintained at (37.0 ± 0.5) °C during all surgical procedures using a feedback-controlled heating pad (BWT-100, Bio Research Center, Nagoya, Aichi, Japan). A polyethylene (PE)-10 tubing (No. 427400; Becton, Dickinson and Company, Franklin Lakes, NJ, USA) was inserted into the tail artery for monitoring of mean arterial blood pressure (MABP) and collection of blood samples. MABP was monitored using a pressure transducer (Edwards Lifesciences, Irvine, CA, USA) and carrier amplifier (AP-601G; Nihon Kohden, Tokyo, Japan), and blood samples were collected using a capillary tube (Radiometer, Copenhagen, Denmark) before ischemia, 30 min after induction of MCAO, and 10 min after reperfusion for measurement of pH, pCO_2_, pO_2_, HCO_3_^−^, and base excess (BE) with a blood gas analyzer (ABL5; Radiometer). The left CCA was exposed through a midline neck incision and tagged with a suture. A 1.5-cm vertical skin incision was made between the left eye and ear. The temporal muscle was scraped and its temperature was monitored with a needle microprobe thermometer and maintained at 37.0 ± 0.5 °C using a heating lamp (TCAT-2AC; Physitemp, Clifton, NJ, USA). Using a small dental drill and fine-tipped rongeur (No. 16021-14; Fine Science Tools, North Vancouver, British Columbia, Canada), a 5 × 7-mm temporal craniotomy was created. The dura mater was then cut by a 27-gauge needle and the distal part of the MCA was occluded immediately lateral to the rhinal fissure using a microclip (MH-1-20; Bear Medic, Tokyo, Japan) with clip applier (No. 11B; Ohwa Tsusho, Tokyo, Japan). The left CCA was soon occluded using the same kind of clip to start the ischemic period. Both a reduction in regional cerebral blood flow (CBF) and visual absence of blood flow were confirmed. After 60 min, both clips were removed and reperfusion was confirmed by CBF measurement and also visually, then the wounds were closed. Rectal temperature was monitored during recovery from anesthesia, then rats were returned to their cages and given access to water. Rectal temperature measured at 30 min, 3, and 24 h after completing 60 min of ischemia in order to ascertain whether rats were not hypothermia. Sham-operated rats underwent the same anesthesia and surgical procedure without MCA/CCAO. Histological assessment (immunostaining and cell counting) and infarct volume measurement were performed by investigators blinded to experimental group assignments.

### Measurement of regional CBF

Regional CBF (rCBF) was monitored before ischemia, 30 min after induction of MCAO, and 10 min after reperfusion in the cerebral cortex of the left hemisphere in the territory supplied by the MCA using a laser Doppler flowmeter (ALF21; Advance, Tokyo, Japan). The laser Doppler probe (CS; Advance) was placed on the brain surface just below where the linea temporalis crossed the MCA. Regional CBF was expressed as a percentage of the pre-ischemia baseline value. Rats that did not show rCBF reduction to <20% during ischemia or >50% after reperfusion were excluded from further examination.

### Post-ischemic anti-ORAIP mAb treatment

Prior to tMCAO surgery, a sterilized 25 µL-syringe (Hamilton, Reno, NV, USA) and the ALZET brain infusion kit 2 (Durect) were assembled and filled with antibody. The kit was implanted into the left lateral ventricle and affixed to the skull, as described above. The tMCAO procedure was then performed. Immediately after reperfusion, a 24-µL volume was infused through the kit into the left lateral ventricle over the course of 30 min (288 μg/h). After this injection, the kit was kept in place for 15 min, then slowly withdrawn to prevent reflux. For post-treatment study, because ORAIP secretion reached a peak level shortly after reperfusion (up to 30 min), we administered the anti-ORAIP mAb for only 30 min. Therefore, to equalize the total amounts of ORAIP mAb between pre-treatment (2 μg/h for 73 h) and post-treatment studies, we determined the dose for post-treatment study at (288 μg/h for 30 min).

### Collection of CSF and plasma samples

CSF and blood samples were collected to determine concentrations of ORAIP in all groups pre-ischemia, 60 min after induction of MCAO (just before reperfusion), and 15 min, 30 min, 60 min, 2 h, 3 h, and 6 h after reperfusion. Devices for withdrawing CSF samples were created as described previously^53^, with minor modifications. Briefly, these devices comprised two 23-gauge needles and connecting and covering them with a 30-cm-long PE-50 tube (No. 427410; Becton, Dickinson and Company) and a 1-mL syringe. Rats were randomly assigned to each time-point group. The rats were positioned prone and the cisterna magna was exposed with minimum bleeding after dissecting the suboccipital muscles and resecting the atlanto-occipital ligament. Next, tMCAO surgery was performed as previously described. The dura was punctured horizontally and centrally to guide the needle into the cisterna magna while avoiding brain parenchyma. Gentle aspiration with the 1-mL syringe enabled collection of about 80 µL of colorless CSF. CSF samples contaminated by blood as shown by sample discoloration were excluded. At the same time, a 1-mL blood sample was collected from the tail artery and centrifuged at 8,000 rpm for 5 min, to obtain a 400-µL clear plasma sample. Plasma samples showing evidence of hemolysis were excluded. Both samples were collected once from each rat and stored at −80 °C for later ELISA, then rats were euthanized by isoflurane overdose.

### Evaluation of infarct volume by 2,3,5-triphenyltetraolium chloride (TTC) staining

Rats were sacrificed under deep anesthesia at 24 h after reperfusion. Brains were quickly removed, frozen at −80 °C, and sectioned into 2-mm coronal slices in acrylic rat brain slicer matrix (RBS-02C; Muromachi Kikai, Tokyo, Japan). Slices were stained with 2% TTC (Sigma-Aldrich, St Louis, MO, USA) in PBS for 15 min at 37 °C in the dark, then fixed with 10% formaldehyde neutral-buffered solution (Sigma-Aldrich) overnight at 4 °C prior to analysis. Infarct volume was measured using ImageJ software in a blinded manner. To compensate for the effects of brain edema in the ischemic area, infarct volume was calculated by subtracting the ipsilateral non-ischemic area from that of the whole contralateral hemisphere, and multiplying the result by slice thickness (2 mm), then calculating the sum for 8 slices.

### Immunohistochemistry and cell counting

Brains were removed and 20-µm-thick brain sections were prepared for immunofluorescent examination. Immunohistochemical and TUNEL staining were performed on freshly frozen coronal sections at the level of the anterior commissure. Frozen sections were fixed with methanol for 10 min at −20 °C, followed by 1% bovine serum albumin (BSA) (A2153; Sigma-Aldrich) in PBS blocking for 60 min at 37 °C, and avidin biotin solution (SP-2001; Vector Laboratories, Burlingame, CA, USA) for 15 min at 37 °C. Fluorescence images were acquired using fluorescence microscopy (BZ-9000; Keyence, Osaka, Japan) and analyzed semi-quantitatively with ImageJ. For cell counting, average staining intensity was calculated from 3 randomly selected microscopic fields in the area of the ischemic penumbra in each brain. For fluorescence analysis, region of interests (ROIs) were randomly set in the areas of penumbra (cortex, striatum) and ischemic core. The penumbral region was defined as the area of the ischemic region spared by pre-ischemic anti-ORAIP mAb treatment in this study, and the ischemic core region was defined as the area of ischemic region that was not spared (Supplemental Fig. 2 in the online-only Data Supplement).

### Cells and hypoxia/reoxygenation

Isolation of cerebral neurons from neonatal rat cerebrum was performed using the SUMITOMO Nerve-Cell Culture System (DS Pharma Biomedical, Osaka, Japan; according to the instructions from the manufacturer). Cells were cultured for 10 days until confluence. Hypoxic conditions (95% N_2_, 5% CO_2_, <0.1% O_2_) were generated as described previously^54^. After incubating cells under hypoxic conditions for the indicated time periods, cells were reoxygenated by immediately replacing the hypoxic medium with normoxic medium for the indicated time periods. Because we found that 10 μg/mL of ORAIP induced apoptosis in up to 50–70% of rat cardiac myocytes^28^ and murine pancreatic β-cells^31^ in 48 h, we chose the dose of 10 μg/mL as a necessary and sufficient dose to induce apoptosis in neurons in the present study.

### Anti-eIF5A mAbs

Mouse anti-eIF5A mAb (clone YSP5-45-36) was generated against human eIF5A peptides (amino acid residues 44–72, including the hypusination site and 69^th^ tyrosine sulfation site, coupled to KLH). Another mouse anti-eIF5A mAb (clone YSPN2-74-18) was generated against human eIF5A peptides (amino acid residues 7–33, near the N-terminal region, coupled to KLH) as described previously^28^.

### Recombinant-ORAIP (re-ORAIP)

Re-ORAIP protein was prepared as described previously^28^. Briefly, FLAG- and His-tagged eIF5A expression vector was transfected into a quail muscle cell line (CRL-1962, American Type Culture Collection). Forty-eight hours after transfection, the cells were subjected to 20 min hypoxia followed by 10 min reoxygenation in PBS. The reoxygenation-conditioned PBS was collected and concentrated, then, the re-ORAIP was purified with Ni-NTA Purification System (Invitrogen), followed by two-step gel filtration chromatography.

### Western blot analysis

Western blot analysis for phosphorylation of ERK1/2 were performed in the same way as cardiac myocytes^28^. Briefly, cultured neurons were subjected to hypoxia/reoxygenation for the indicated time periods, then the cells were frozen in liquid nitrogen, lysed with cell lysis buffer (Cell Signaling Technology [CST]) and centrifuged. The supernatants were suspended in Laemmli’s sample buffer, then subjected to Western blot analysis using a rabbit polyclonal phospho-specific anti-ERK1/2 (Thr202/Tyr204) and control anti-ERK1/2 antibodies (CST).

### ELISA

Sandwich ELISA was performed using YSPN2-74-18 as a capture antibody fixed on the wells of microtiter strips. CSF or plasma samples were pipetted into the wells and incubated. After washing, horseradish peroxidase (HRP)-labeled YSP5-45-36 was added as a detection antibody and incubated. After washing, color development was carried out by addition of a substrate solution, as described previously^28^.

### Immunofluorescence

Immunofluorescent staining of ORAIP was performed using tyramide signal amplification (TSA) technology for fluorescence (TSA^TM^ Biotin System; PerkinElmer, Waltham, MA, USA). Double-immunostaining for neuron-specific markers such as NeuN or NSE antigens was performed as described elsewhere^55^. For double-immunostaining for ORAIP and NeuN, cells were fixed in acetone for 5 min for cultured cerebral neurons, or in methanol for 10 min for *in vivo* sections, and were first incubated with HRP-labeled anti-ORAIP mAb (YSP5-45-36, 5 µg/ml), followed by incubation with biotinylated tyramide, then with fluorescein-avidin D. Cells were then incubated with rabbit anti-NeuN (1:200, ABN78; Millipore, Temecula, CA, USA) followed by incubation with tetramethylrhodamine isothiocyanate (TRITC)-labeled anti-rabbit IgG (1:200, T6778; Sigma-Aldrich). To stain for Annexin-V, cells were incubated in biotinylated Annexin-V in 1× binding buffer (Annexin V-Biotin Apoptosis Detection Kit; BioVision, Milpitas, CA, USA) for 5 min, then fixed with 2% paraformaldehyde in PBS for 15 min. Double-immunostaining for the neuron-specific antigen NSE was performed using mouse anti-NSE mAb (NA 1501, clone 47; Enzo Life Sciences, Farmingdale, NY, USA) and TRITC-labeled anti-mouse IgG. Immunofluorescent staining of tissue samples was performed as for cultured cells. Double-immunostaining for glial cells and oligodendrocytes was performed using rabbit anti-GFAP (1:150, ABN5804; Millipore) and rabbit anti-OLIG2 (1:50, AB9610; Millipore) antibodies, respectively. Immunofluorescent staining with mouse IgG instead of first antibodies was done as negative controls.

### TUNEL staining

We also used the *In Situ* Apoptosis Detection Kit (TAKARA BIO, Kusatsu, Japan for cultured cells, or Roche Diagnostics, Indianapolis, IN, USA for *in vivo* frozen sections) followed by diaminobenzidine reaction (brown color) for TUNEL staining. For additional neuron-specific double-immunostaining, cells were incubated with anti-NeuN antibody (1:200, ABN78; Millipore) followed by alkaline phosphatase-labeled anti-rabbit IgG (1:200, T6778; Sigma-Aldrich). Cells were then reacted with an alkaline phosphatase substrate (alkaline phosphatase substrate kit III; Vector Laboratories) to produce a blue reaction product.

### Statistical analysis

All data are presented as mean ± standard error of the mean (s.e.m.) or standard deviation (s.d.). Comparisons between two groups were performed using Welch’s *t*-test. For multiple comparisons, one-way analysis of variance (ANOVA) was followed by Tukey-Kramer analysis unless otherwise indicated. Values of *P* < 0.05 were considered statistically significant.

## Supplementary information

Supplementary Material
