# Supplementary material for: Oxidative stress-responsive apoptosis inducing protein (ORAIP) plays a critical role in cerebral ischemia/reperfusion injury
Source: Sci Rep. 2019 Sep 18;9:13512. doi: 10.1038/s41598-019-50073-8 (PMC6751213; doi:10.1038/s41598-019-50073-8)
Supplement: Supplementary file 1 — Supplementary Material [file 41598_2019_50073_MOESM1_ESM.docx]

**ONLINE SUPPLEMENTAL MATERIAL**

**Oxidative stress-responsive apoptosis inducing protein (ORAIP) plays a critical role in cerebral ischemia/reperfusion injury**

Masao Kishimoto,^1^ MD; Jun Suenaga,^1^ MD, PhD; Hajime Takase,^1^ MD, PhD;

Kota Araki,^1^ MD; Takako Yao,^2^ ;Tsutomu Fujimura,^3^ PhD; Kimie Murayama,^4^ PhD;

Ko Okumura,^5^ MD, PhD; Ryu Ueno,^1^ MD; Nobuyuki Shimizu,^1^ MD, PhD;

Nobutaka Kawahara,^1†^ MD, PhD, Tetsuya Yamamoto,^1^ MD, PhD; Yoshinori Seko,^5^ MD, PhD

^1^Department of Neurosurgery, Yokohama City University Graduate School of Medicine, Yokohama, Japan; ^2^Division of Cardiovascular Medicine, The Institute for Adult Diseases, Asahi Life Foundation, Tokyo, Japan; ^3^Laboratory of Bioanalytical Chemistry, Tohoku Medical and Pharmaceutical University, Sendai, Japan; ^4^Division of Proteomics and Biomolecular Science, BioMedical Research Center, Graduate School of Medicine, Juntendo University, Tokyo, Japan; ^5^Department of Biofunctional Microbiota, Juntendo University School of Medicine, Tokyo, Japan.

† Deceased 14 May, 2016

**Corresponding author:** Jun Suenaga, MD, PhD,

Department of Neurosurgery, Yokohama City University Graduate School of Medicine,

3-9 Fukuura, Kanazawa-ku, Yokohama, Kanagawa, Japan 236-0004

E-mail: suenaga@yokohama-cu.ac.jp

TEL: +81-45-787-2663 FAX: +81-45-783-6121

**Abbreviations:** AIF, apoptosis-inducing factor; AIS, acute ischemic stroke; ANOVA, analysis of variance; BBB, blood-brain barrier; BE, base excess; CBF, cerebral blood flow ; CCAO, common carotid artery occlusion; CpG ODN, cytosine-guanine oligodeoxynucleotide; CSF, cerebrospinal fluid; eIF5A, eukaryotic translation initiation factor 5A; ELISA, enzyme-linked immunosorbent assay; ERK, extracellular signal-regulated kinase; FITC, fluorescein isothiocyanate; GFAP, glial fibrillary acidic protein; HRP, horseradish peroxidase; ICV, intracerebroventricular; I/R, ischemia/reperfusion; mAb, monoclonal antibody; MABP, mean arterial blood pressure; MCAO, middle cerebral artery occlusion; NeuN, neuronal nuclei; NSE, neuron-specific enolase; OLIG2, oligodendrocyte transcription factor 2; ORAIP, oxidative stress-responsive apoptosis inducing protein; PBS, phosphate-buffered saline; PE, polyethylene; re, recombinant; RNPs, nitroxide radical-containing nanoparticles; ROI, region of interest; ROS, reactive oxygen species; s.d., standard deviation; s.e.m., standard error of the mean; SHR, spontaneously hypertensive rat; tPA, tissue plasminogen activator; TRITC, trimethylrhodamine isothiocyanate; TSA, tyramide signal amplification; TTC, 2,3,5-triphenyltetraolium chloride; TUNEL, terminal deoxynucleotidyl transferase-mediated deoxyuridine triphosphate nick-end labeling.

**Supplemental Tables**

| Groups (number of rats) | | Parameters | |
| --- | --- | --- | --- |
|  |  | Age (weeks) | Body weight (g) |
| PBS (8) | | 12.3 ± 0.9 | 290.9 ± 12.2 |
| mouse IgG 6 μg/h (9) | | 11.9 ± 0.9 | 292.3 ± 13.7 |
| anti-ORAIP mAb 2 μg/h (10) | | 12.0 ± 0.9 | 293.5 ± 14.2 |
| anti-ORAIP mAb 6 μg/h (9) |  | 11.8 ± 0.6 | 281.7 ± 14.5 |

**Supplemental Table I. Age and body weight in pre-treatment experimental groups.**

Age and body weight were measured at intraventricular injection. No significant differences between groups were identified for any parameters (ANOVA). All values are expressed as mean ± s.d.

| Parameters | Groups (number of rats) | Pre-ischemia | During ischemia | Post-ischemia |
| --- | --- | --- | --- | --- |
| pH | PBS (8) | 7.42 ± 0.03 | 7.43 ± 0.03 | 7.42 ± 0.02 |
|  | mouse IgG 6 μg/h (9) | 7.43 ± 0.02 | 7.43 ± 0.01 | 7.43 ± 0.02 |
|  | anti-ORAIP mAb 2 μg/h (10) | 7.42 ± 0.02 | 7.42 ± 0.03 | 7.42 ± 0.02 |
|  | anti-ORAIP mAb 6 μg/h (9) | 7.42 ± 0.03 | 7.42 ± 0.02 | 7.42 ± 0.02 |
| PaCO_2_ (mm Hg) | PBS (8) | 46.4 ± 4.9 | 39.1 ± 3.7 | 39.8 ± 3.7 |
|  | mouse IgG 6 μg/h (9) | 42.9 ± 3.2 | 40.4 ± 2.3 | 39.1 ± 2.6 |
|  | anti-ORAIP mAb 2 μg/h (10) | 42.8 ± 2.7 | 40.5 ± 4.1 | 38.9 ± 3.5 |
|  | anti-ORAIP mAb 6 μg/h (9) | 43.6 ± 5.7 | 38.7 ± 3.5 | 38.0 ± 3.0 |
| PaO_2_ (mm Hg) | PBS (8) | 155.9 ± 39.8 | 140.5 ± 17.4 | 139.5 ± 19.0 |
|  | mouse IgG 6 μg/h (9) | 148.0 ± 15.6 | 139.9 ± 36.2 | 133.2 ± 16.9 |
|  | anti-ORAIP mAb 2 μg/h (10) | 154.1 ± 24.3 | 142.2 ± 18.6 | 132.4 ± 21.3 |
|  | anti-ORAIP mAb 6 μg/h (9) | 144.1 ± 17.5 | 149.1 ± 10.5 | 145.9 ± 18.3 |
| HCO_3_^-^ (mmol/L) | PBS (8) | 29.5 ± 1.8 | 25.1 ± 2.2 | 25.1 ± 1.6 |
|  | mouse IgG 6 μg/h (9) | 27.9 ± 2.1 | 26.4 ± 1.3 | 26.0 ± 1.1 |
|  | anti-ORAIP mAb 2 μg/h (10) | 27.4 ± 1.3 | 26.0 ± 2.0 | 24.9 ± 1.8 |
|  | anti-ORAIP mAb 6 μg/h (9) | 27.8 ± 2.0 | 25.3 ± 2.0 | 24.5 ± 1.3 |
| BE (mmol/L) | PBS (8) | 4.5 ± 1.6 | 1.4 ± 2.1 | 1.4 ± 1.4 |
|  | mouse IgG 6 μg/h (9) | 3.4 ± 1.9 | 2.7 ± 1.2 | 2.1 ± 1.1 |
|  | anti-ORAIP mAb 2 μg/h (10) | 2.9 ± 1.1 | 1.7 ± 1.7 | 0.9 ± 1.4 |
|  | anti-ORAIP mAb 6 μg/h (9) | 3.3 ± 1.4 | 1.6 ± 1.3 | 0.6 ± 1.1 |

**Supplemental Table II. Blood gas analysis data in pre-treatment experimental groups.**

During-ischemia and post-ischemia data were obtained at 30 min after induction of ischemia and at 10 min after reperfusion, respectively. No significant differences in any parameters were seen among groups (ANOVA). All values are expressed as mean ± s.d.

| Parameters | Groups (number of rats) | Pre-ischemia | During ischemia | Post-ischemia |  |  |  |  |
| --- | --- | --- | --- | --- | --- | --- | --- | --- |
| MABP | PBS (8) | 129.7 ± 13.7 | 129.3 ± 14.2 | 145.8 ± 11.7 |  |  |  |  |
| (mm Hg) | mouse IgG 6 μg/h (9) | 126.2 ± 15.4 | 141.7 ± 21.5 | 135.4 ± 23.5 |  |  |  |  |
|  | anti-ORAIP mAb 2 μg/h (10) | 130.6 ± 13.8 | 137.5 ± 17.6 | 146.1 ± 20.2 |  |  |  |  |
|  | anti-ORAIP mAb 6 μg/h (9) | 123.9 ± 21.7 | 126.7 ± 12.5 | 140.9 ± 15.1 |  |  |  |  |
| TT (°C) | PBS (8) | 37.1 ± 0.4 | 37.0 ± 0.4 | 37.3 ± 0.4 |  |  |  |  |
|  | mouse IgG 6 μg/h (9) | 37.1 ± 0.3 | 36.7 ± 0.3 | 37.1 ± 0.4 |  |  |  |  |
|  | anti-ORAIP mAb 2 μg/h (10) | 37.1 ± 0.4 | 36.9 ± 0.2 | 37.0 ± 0.3 |  |  |  |  |
|  | anti-ORAIP mAb 6 μg/h (9) | 37.1 ± 0.3 | 36.8 ± 0.3 | 37.1 ± 0.3 |  |  |  |  |
| RT (°C) | PBS (8) | 37.3 ± 0.3 | 37.1 ± 0.3 | 37.2 ± 0.3 |  |  |  |  |
|  | mouse IgG 6 μg/h (9) | 37.2 ± 0.3 | 37.0 ± 0.2 | 37.1 ± 0.3 |  |  |  |  |
|  | anti-ORAIP mAb 2 μg/h (10) | 37.2 ± 0.3 | 37.2 ± 0.3 | 37.2 ± 0.3 |  |  |  |  |
|  | anti-ORAIP mAb 6 μg/h (9) | 37.3 ± 0.3 | 37.2 ± 0.2 | 37.1 ± 0.2 |  |  |  |  |
| Lt. CBF | PBS (8) | 51.9 ± 9.1 | 4.1 ± 2.0 | 49.2 ± 9.9 |  |  |  |  |
| (ml/min/100g) | mouse IgG 6 μg/h (9) | 52.6 ± 9.3 | 3.9 ± 1.1 | 50.7 ± 13.9 |  |  |  |  |
|  | anti-ORAIP mAb 2 μg/h (10) | 52.9 ± 4.6 | 4.8 ± 2.4 | 52.6 ± 14.0 |  |  |  |  |
|  | anti-ORAIP mAb 6 μg/h (9) | 49.4 ± 5.8 | 4.1 ± 2.1 | 52.3 ± 6.9 |  |  |  |  |
| **Supplemental Table III. Physiological parameters in pre-treatment experimental groups.**  MABP=mean arterial blood pressure; TT=temporal muscle temperature; RT=rectal temperature; CBF=cerebral blood flow. | | | | | | |  |  |
| During-ischemia and post-ischemia data were obtained at 30 min after induction of ischemia and at 10 min after reperfusion, respectively. No significant differences between groups were seen for any parameters (ANOVA). All values are expressed as mean ± s.d. | | | | | |  |  |  |
|  | | | | | | | | |

| Parameter | Groups (number of rats) | 30 min | 3 h | 24 h |
| --- | --- | --- | --- | --- |
| RT (°C) | PBS (8) | 37.2 ± 0.3 | 37.4 ± 0.5 | 37.6 ± 0.3 |
|  | mouse IgG 6 μg/h (9) | 37.3 ± 0.3 | 37.5 ± 0.3 | 37.5 ± 0.2 |
|  | anti-ORAIP mAb 2 μg/h (10) | 37.3 ± 0.3 | 37.3 ± 0.3 | 37.5 ± 0.2 |
|  | anti-ORAIP mAb 6 μg/h (9) | 37.1 ± 0.3 | 37.3 ± 0.3 | 37.3 ± 0.2 |

**Supplemental Table IV. Rectal temperature after reperfusion in pre-treatment experimental groups.**

Rectal temperature (RT) was measured at 30 min, 3, and 24 h after completing 60 min of ischemia. No significant differences between groups were seen in any parameters (ANOVA). All values are expressed as mean ± s.d.

| Groups (number of rats) | Parameters | |
| --- | --- | --- |
|  | Age (weeks) | Body weight (g) |
| mouse IgG 288 μg/h (8) | 12.5 ± 1.4 | 304.3 ± 14.4 |
| anti-ORAIP mAb 288 μg/h (7) | 12.7 ± 1.4 | 300.6 ± 8.4 |
| **Supplemental Table V. Age and body weight in post-treatment experimental groups.** | | |
| Age and body weight were measured before surgery. | |  |
| No significant differences between groups were seen for any parameters (t-test). | | |
| All values are expressed as mean ± s.d. | |  |

| \| Parameters \| Groups (number of rats) \| Pre-ischemia \| During ischemia \| Post-ischemia \| \| --- \| --- \| --- \| --- \| --- \| \| pH \| mouse IgG 288 μg/h (8) \| 7.43 ± 0.02 \| 7.44 ± 0.03 \| 7.44 ± 0.03 \| \|  \| anti-ORAIP mAb 288 μg/h (7) \| 7.43 ± 0.01 \| 7.42 ± 0.02 \| 7.41 ± 0.02 \| \| PaCO_2_ \| mouse IgG 288 μg/h (8) \| 46.0 ± 3.3 \| 41.1 ± 2.9 \| 40.0 ± 2.7 \| \| (mm Hg) \| anti-ORAIP mAb 288 μg/h (7) \| 44.1 ± 1.2 \| 43.1 ± 2.7 \| 42.0 ± 3.3 \| \| PaO_2_ (mm Hg) \| mouse IgG 288 μg/h (8) \| 151.4 ± 16.4 \| 151.5 ± 14.3 \| 155.3 ± 15.6 \| \|  \| anti-ORAIP mAb 288 μg/h (7) \| 151.0 ± 16.2 \| 160.4 ± 9.8 \| 162.4 ± 8.9 \| \| HCO_3_^-^ \| mouse IgG 288 μg/h (8) \| 29.5 ± 1.4 \| 27.6 ± 1.7 \| 27.0 ± 1.5 \| \| (mmol/L) \| anti-ORAIP mAb 288 μg/h (7) \| 29.3 ± 1.1 \| 27.9 ± 1.6 \| 26.3 ± 1.5 \| \| BE (mmol/L) \| mouse IgG 288 μg/h (8) \| 4.9 ± 1.1 \| 3.5 ± 1.4 \| 2.9 ± 1.7 \| \|  \| anti-ORAIP mAb 288 μg/h (7) \| 4.7 ± 1.1 \| 3.1 ± 1.5 \| 1.9 ± 1.3 \|   **Supplemental Table VI. Blood gas analysis data in post-treatment experimental groups.** |  |  |  |  |  |  |
| --- | --- | --- | --- | --- | --- | --- | --- | --- | --- | --- | --- | --- | --- | --- | --- | --- | --- | --- | --- | --- | --- | --- | --- | --- | --- | --- | --- | --- | --- | --- | --- | --- | --- | --- | --- | --- | --- | --- | --- | --- | --- | --- | --- | --- | --- | --- | --- | --- | --- | --- | --- | --- | --- | --- | --- | --- | --- | --- | --- | --- | --- |
| During-ischemia and post-ischemia data were obtained at 30 min after induction of ischemia and at 10 min after reperfusion, respectively. | | |  |  |  |  |
| No significant differences between groups were identified in any parameters (t-test). | | | | | |  |
| All values are expressed as mean ± s.d. | | | |  |  |  |

| Parameters | Groups (number of rats) | Pre-ischemia | During ischemia | Post-ischemia |
| --- | --- | --- | --- | --- |
| MABP | mouse IgG 288 μg/h (8) | 140.9 ± 21.5 | 129.3 ± 16.5 | 122.6 ± 17.2 |
| (mm Hg) | anti-ORAIP mAb 288 μg/h (7) | 130.3 ± 15.0 | 144.3 ± 14.6 | 123.3 ± 8.1 |
| TT (°C) | mouse IgG 288 μg/h (8) | 36.9 ± 0.3 | 36.8 ± 0.2 | 37.0 ± 0.4 |
|  | anti-ORAIP mAb 288 μg/h (7) | 36.7 ± 0.1 | 36.8 ± 0.2 | 36.9 ± 0.3 |
| RT (°C) | mouse IgG 288 μg/h (8) | 37.2 ± 0.3 | 36.9 ± 0.3 | 37.0 ± 0.3 |
|  | anti-ORAIP mAb 288 μg/h (7) | 36.9 ± 0.3 | 36.9 ± 0.3 | 37.1 ± 0.2 |
| Lt. CBF | mouse IgG 288 μg/h (8) | 52.6 ± 6.3 | 4.2 ± 1.7 | 48.2 ± 9.9 |
| (ml/min/100g) | anti-ORAIP mAb 288 μg/h (7) | 51.1 ± 1.9 | 4.8 ± 1.7 | 43.5 ± 4.5 |
| **Supplemental Table VII. Physiological parameters in post-treatment experimental groups.** | | | | |
| MABP=mean arterial blood pressure; TT=temporal muscle temperature; RT=rectal temperature; CBF=cerebral blood flow. | | | | |
| During ischemia and post-ischemia data were obtained at 30 min after induction of ischemia and at 10 min after reperfusion, respectively. | | | | |
| No significant differences between groups were seen for any parameters (t-test). | | | | |
| All values are expressed as mean ± s.d. | | | | |

| Parameters | Groups (number of rats) | 30 min | 3 h | 24 h |  |
| --- | --- | --- | --- | --- | --- |
| RT (°C) | mouse IgG 288 μg/h (8) | 37.0 ± 0.4 | 37.4 ± 0.5 | 37.5 ± 0.7 |  |
|  | anti-ORAIP mAb 288 μg/h (7) | 37.1 ± 0.2 | 37.4 ± 0.4 | 37.5 ± 0.7 |  |
| **Supplemental Table VIII. Rectal temperature after reperfusion in post-treatment experiments.** | | | | | |
| Rectal temperature (RT) was measured at 30 min, 3, and 24 h after 60 min ischemia. | | | | | |
| No significant differences between groups were seen in any parameters (ANOVA). | | | | | |
| All values are expressed as mean ± s.d. | |  |  |  | |

| Parameters | Groups (number of rats) | Value |  |  |  |
| --- | --- | --- | --- | --- | --- |
| Grip  (% compared to baseline) | PBS (8)  mouse IgG 6 μg/h (9)  anti-ORAIP mAb 2 μg/h (10)  anti-ORAIP mAb 6 μg/h (9) | 65.3 ± 25.2  69.5 ± 47.5  54.7 ± 27.8  66.2 ± 30.6 |  |  |  |
| Bederson scale  (point as baseline defined 0) | PBS (4)  mouse IgG 6 μg/h (3)  anti-ORAIP mAb 2 μg/h (5)  anti-ORAIP mAb 6 μg/h (4) | 1.8 ± 1.3  2.0 ± 0.0  1.4 ± 0.6  1.8 ± 0.5 |  |  |  |
| **Supplemental Table IX. Behavior tests at 24 h after reperfusion in pre-treatment experimental groups.** | | | | | |
| Grip motor function measured by dynamometer or Bederson scale composed of forelimb flexion, resistance to lateral push and circling behavior were tested at 24 h after reperfusion. Rats are exposed to 60 min tandem MCA occlusion under 73 h pre-treatment of PBS, IgG, or anti-ORAIP mAbs. No significant differences between groups were seen in any parameters (ANOVA). All values are expressed as mean ± s.d. | | | | | |
|  | | | | | |
|  | |  |  |  | |

**Supplemental Figures**


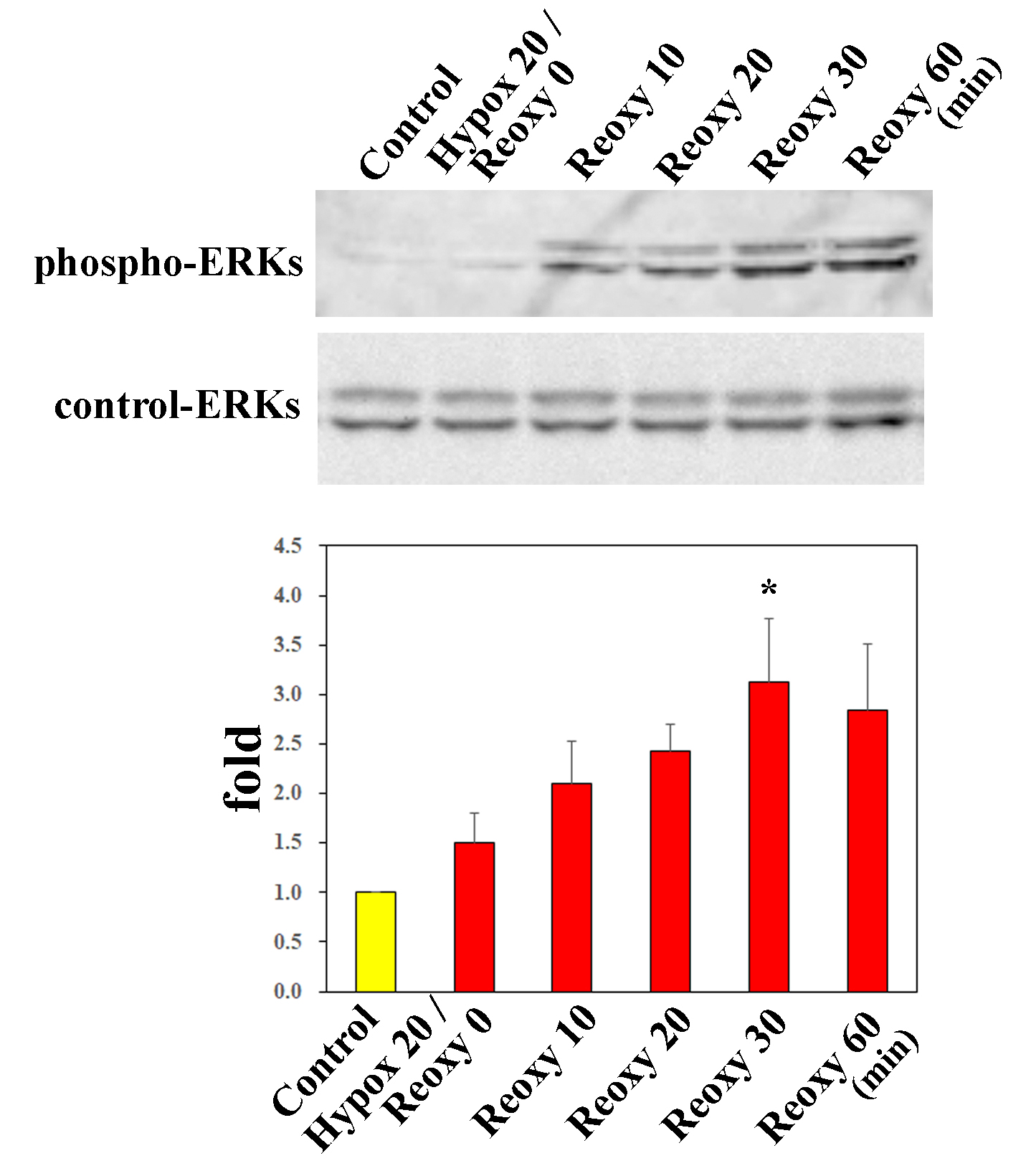


**Supplemental Figure I. Hypoxia/reoxygenation activates ERKs in cerebral neurons *in vitro*.**

Hypoxia (20 min)/reoxygenation significantly activated ERKs, reaching the peak level by 30 min after reoxygenation (Hypox 20/Reoxy 30) ([mean ± s.e.m.] n=5, **P*=0.0059 vs, Control, **P*=0.0348 vs Hypox 20/Reoxy 0). There were no significant differences between Hypox 20/Reoxy 30 and Hypox 20/Reoxy 10, 20, and 60. (Dunnett’s multiple comparison test)..


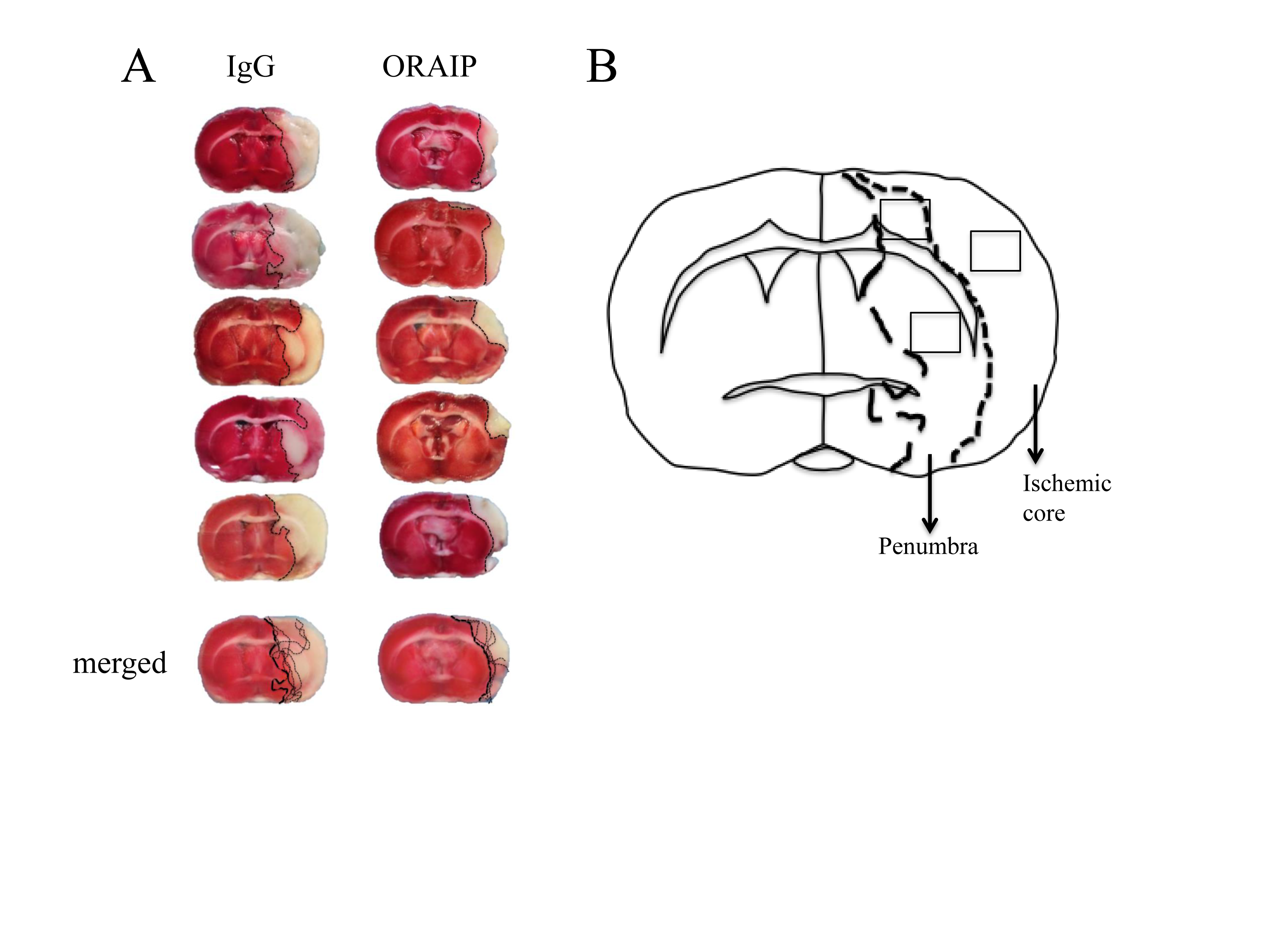


**Supplemental Figure II. Images and schematic showing brain regions at the anterior commissure and concerning ischemic core and penumbra in pre-treatment experiments.**

**A,** Representative images of TTC staining in IgG and ORAIP groups, n=5 for each group. Merged image is sum of them. **B,** Representative schema defined as ischemic core and penumbra. Squares show the region of interests (ROIs). The cells were counted at 3 random microscopic fields in the squares.


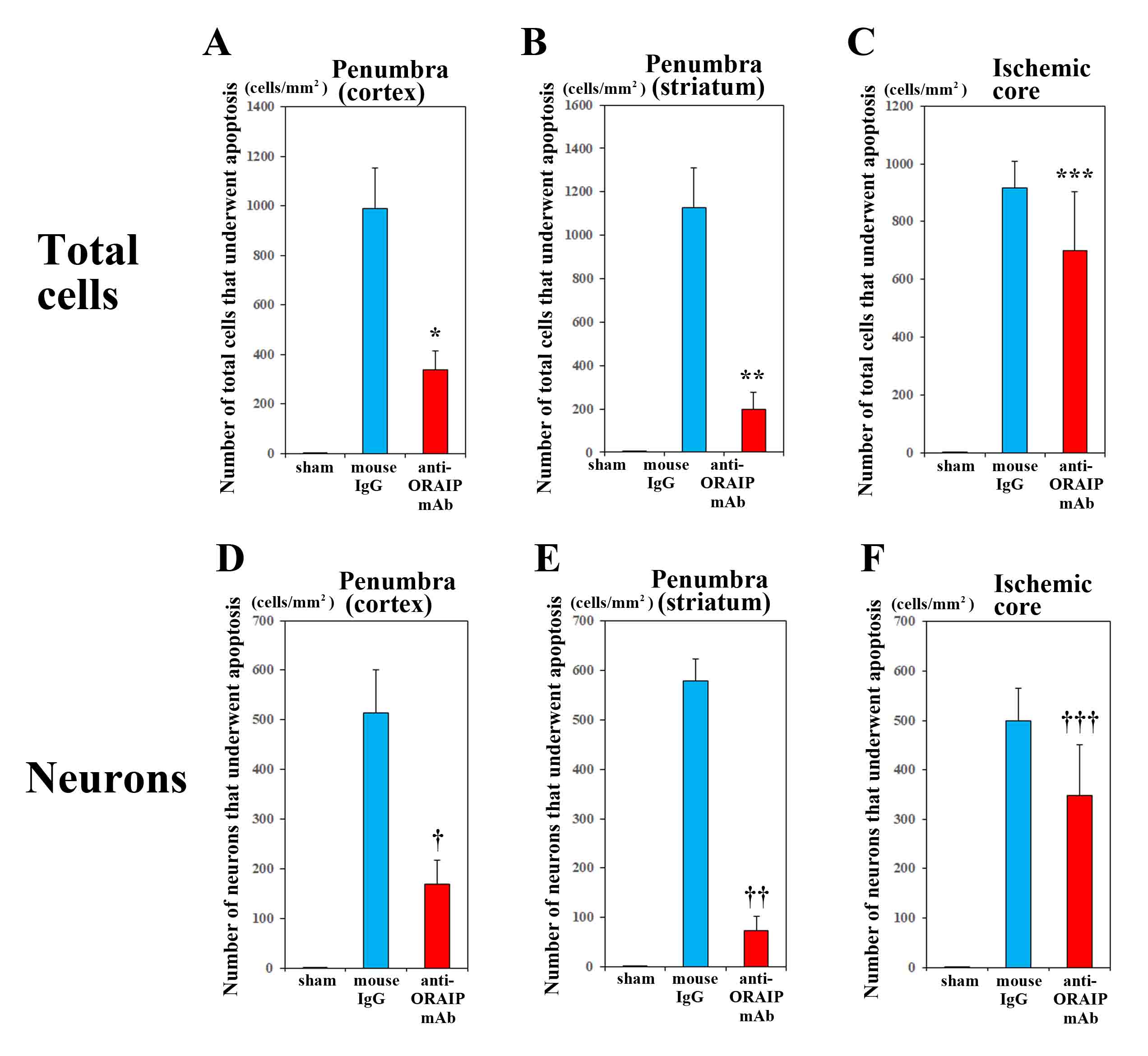
**Supplemental Figure III. Effects of pre-treatment with anti-ORAIP neutralizing mAb on apoptosis induction of total neural cells (panels A-C) and neurons (panels D-F) in the penumbra (cortex and striatum) and ischemic core areas during ischemia (60 min) and after reperfusion (24 h).**

Pre-treatment with anti-ORAIP neutralizing mAb (6 μg/h, 73 h) significantly suppressed apoptosis induction in total neural cells as well as in neurons within the penumbral (cortex and striatum) areas (total neural cells, **P*=0.0012, ***P*<0.001; neurons, †*P*=0.0018, ††*P*<0.001 vs. mouse IgG, respectively). Pre-treatment with anti-ORAIP neutralizing mAb tended to suppress (but not significantly) induction of apoptosis in total neural cells as well as in neurons within the ischemic core area (total neural cells, ****P*=0.4371; neurons, †††*P*=0.3108 vs. mouse IgG). ([mean ± s.e.m.], n=6 for each, Tukey-Kramer method).

| **B**  **A**  **rCBF (ml/min/100g)**  **rCBF (ml/min/100g)**   \|  \|  \|  \| \| --- \| --- \| --- \|  \| Post-  ischemia \|  \|  \| \| --- \| --- \| --- \|  \| Pre-  ischemia \|  \|  \| \| --- \| --- \| --- \|  \| During-  ischemia \|  \|  \| \| --- \| --- \| --- \|  \| Pre-  ischemia \|  \|  \| \| --- \| --- \| --- \|  \| During-  ischemia \|  \|  \| \| --- \| --- \| --- \|  \| Post-  ischemia \|  \|  \| \| --- \| --- \| --- \|   **Supplemental Figure Ⅳ. CBF changes in pre- (A) and post- (B) treatment experimental groups.** |
| --- | --- | --- | --- | --- | --- | --- | --- | --- | --- | --- | --- | --- | --- | --- | --- | --- | --- | --- | --- | --- | --- |
| rCBF= regional cerebral blood flow. |
| During ischemia and post-ischemia data were obtained at 30 min after induction of ischemia and at 10 min after reperfusion, respectively. No significant differences between groups were seen for any parameters (t-test). All values are expressed as mean ± s.e.m. |
|  |
|  |
